# Association of foveal avascular zone (FAZ) enlargement with SARS-CoV-2 infection and long COVID-19

**DOI:** 10.1186/s12886-025-04528-4

**Published:** 2025-11-28

**Authors:** J. Spielmann, I. Pink, C. Framme, J. Tode, I. Volkmann

**Affiliations:** 1 Hannover Medical School, University Eye Hospital, Carl-Neuberg-Street 1, 30625 Hannover, Germany; 2 Department of Pulmonology, General Medicine, Hannover Medical School, Hannover, Germany

**Keywords:** SARS-CoV-2, Long COVID-19, OCTA, Fatigue, FAZ

## Abstract

**Background:**

As COVID-19 was found to affect small vessels of multiple organ systems, the purpose of the study is to describe the macular vessel density (VD) and the area of the foveal avascular zone (FAZ) by optical coherence tomography angiography (OCTA) in patients who recovered from a COVID-19 disease, who were or were not treated in an Intensive Care Unit (ICU) and who suffer from fatigue due to long COVID.

**Methods:**

Prospective case-control study in which laboratory-confirmed and recovered COVID-19 patients were included. Macular OCTA was performed at least seven weeks after initial COVID-19. The VD of the superficial retinal capillary plexus (SCP) and the area of the FAZ were measured. Results were compared among ICU- and non-ICU COVID-19 patients and a healthy control group; moreover, patients were divided into groups of no fatigue, fatigue, and extreme fatigue and results were compared among these and a control group.

**Results:**

FAZ of non-ICU patients was significantly enlarged compared to control group. VD was not significantly different among these cohorts. FAZ of fatigue group was significantly enlarged compared to no fatigue group and control group. VD of extreme fatigue group was significantly greater than VD of both no fatigue group and control group.

**Conclusion:**

The retinal microvasculature of recovered COVID-19 patients showed alterations in the sense of a significantly enlarged FAZ, particularly in patients with mild disease and fatigue. These microvascular manifestations may lead to future complications or visual impairment. Performing macular OCTA could be a possible monitoring tool for microvascular changes in these patients and a predictor for long COVID fatigue.

## Background

The severe acute respiratory syndrome coronavirus type 2 (SARS-CoV-2), that first appeared in Wuhan, China in December 2019, causes the coronavirus disease 2019 (COVID-19) [1]. The World Health Organization (WHO) declared COVID-19 a pandemic on March 11, 2020, due to rapidly rising incidence of COVID-19 all over the world [2]. The number of confirmed cases of COVID-19 increased to more than 621 million by October 16, 2022 [3].

With an increasing number of patients and more research work done worldwide, not only pneumonia but systemic inflammation was soon described to be caused by SARS-CoV-2 [4–6]. The virus was found to enter cells of many human tissues using Angiotensin-converting enzyme 2 (ACE2) as a receptor on cell surfaces [7]. Also damage to the vascular system including endothelial injury and thrombotic events not only in large, but also in microscopic blood vessels, was found to be an important part of the mechanisms of COVID-19 [8, 9], whilst studies describe a link between inflammation and these vascular alterations [10–12].

Patients who already recovered from COVID-19 can suffer from persisting symptoms beyond the acute phase of infection including fatigue as the most common symptom with an estimated global prevalence of 23% among those infected with COVID-19 [13]. Other symptoms can be cognitive impairment and various physical (e.g., dyspnea) manifestations [14–16]. The current guideline of UK’s National Institute of Health and Care Excellence (NICE) summarizes these persisting symptoms to long COVID, which divides into ongoing symptomatic COVID-19 (symptoms of COVID-19 from 4 weeks up to 12 weeks) and post-COVID-19 syndrome (symptoms of COVID-19 for 12 weeks or more) [17]. The estimated global prevalence of the post-COVID-19 syndrome is 43 percent among those infected with COVID-19 [13], which demonstrates its great importance for global health.

COVID-19 affects multiple organ systems [5] and the eyes are affected, as well. Conjunctivitis was repeatedly seen in patients, so COVID-19 was soon thought to implicate the anterior segment [18–20]. But retinal involvement in COVID-19 is also described [21, 22] as ACE2, which SARS-CoV-2 uses to enter host cells, is expressed in multiple cell types in the neuroretina and in the retinal endothelium [23]. In particular, the retinal microvasculature was found to be altered in COVID-19 patients [24–27]. To obtain these results, vessels were analyzed using the non-invasive optical coherence tomography angiography (OCTA), which has become a common tool for evaluating retinal vascular diseases particularly for research purposes over the last few years [28].

The purpose of this study is to generate further data concerning the retinal microvasculature of recovered COVID-19 patients using OCTA, as the review of current evidence by Teo et. al. suggests despite already cumulating evidence of retinal microangiopathy caused by SARS-CoV-2, noting the scale of the potential issue [24]. Also, further information on the findings about long COVID-19 is required, to which we aim to contribute by describing the retinal microvasculature in patients with no fatigue, fatigue, or extreme fatigue after recovering from COVID-19.

## Methods

### Study population

The prospective case-control study was conducted at the ophthalmological department of Hannover Medical School (MHH), Germany between July 2020 and August 2021. It was carried out in accordance with the Declaration of Helsinki, registered in Deutsches Register Klinische Studien (German Clinical Trials Register, Clinical Trial Number: DRKS00022874; Registration date: 20 August 2020) and with approval of the Ethics Committee of MHH (Nr. 9159_BO_K_2020). Written informed consent was given by all participants. Patients who were infected with SARS-CoV-2, which was confirmed at least seven weeks ago by a positive test result with real-time reverse transcription polymerase chain reaction of a nasopharyngeal swab sample, were included. At the time of the examination, the patients were in treatment in the COVID-19 aftercare outpatient clinic of MHH because of long COVID-19 symptoms, of which we focused on fatigue, assessing its severity with the Fatigue Assessment Scale (© FAS Fatigue Assessment Scale: ild care foundation http://www.ildcare.nl) [29]. The patients are divided into two study cohorts depending on whether they were (*n* = 5 patients) or were not (*n* = 35 patients) treated in an intensive care unit due to COVID-19. From healthy subjects with no history of laboratory confirmed SARS-CoV-2 infection, the control group (*n* = 37 patients) was selected. Age (*p* = 0.618) and sex distribution (*p* = 0.297) did not differ statistically significant between the study cohorts.

For consideration of the possible correlation of fatigue and retinal microvasculature, the SARS-CoV-2 confirmed subjects were divided into three cohorts: No fatigue (*n* = 14; score of < 22 on FAS), fatigue (*n* = 17; < 35 on FAS) and extreme fatigue (*n* = 6; ≥ 35 on FAS). These three groups were compared to a control group that includes the subjects with no history of laboratory confirmed SARS-CoV-2 infection.

Patients with a history of previous retinal damage as macular edema, glaucoma, vasculitis, or other degenerative alterations were excluded from this study.

Patients with diabetes or cardiovascular comorbidities were not excluded from the study.

The best corrected visual acuity (BCVA) and refraction was evaluated using a Topcon KR-800S Auto Kerato Refractometer (Topcon Corporation, Japan). For intraocular pressure measurements, the Nidek NT-510 non-contact-tonometer (NIDEK CO., LTD., Japan) was used.

### OCTA image acquisition and analysis

For all OCTA scans, a SPECTRALIS® OCT (Heidelberg Engineering, Germany) was used. A 3 × 3 mm scan of the macula was performed. OCTA images of the Superficial Capillary Plexus (SCP) were used to measure vessel density (VD) and foveal avascular zone (FAZ) area. OCTA images deemed unsuitable due to artifacts were excluded; the selection was performed manually by the author. No masked review or adjudicated assessment was performed.

Analysis of OCTA images were performed with the image processing software ImageJ (Version 1.53a, National Institutes of Health, USA). The OCTA images were automatically binarized using the “Make binary” function of ImageJ (Process drop-down menu), and the vascular area, marked as white pixels, was measured in pixels/mm^2^. FAZ area (mm^2^) was measured manually by marking the avascular zone of the binarized image as a polygon using “polygon selections” function and calculating the marked area with “measure” function of ImageJ.

### Statistical analysis

For statistical analysis, the statistical software SPSS (Version 27, IBM, New Castle, NY, USA) was used. The Shapiro-Wilk-Test was used to examine the distribution of variables and homogeneity of variances was asserted using Levene’s Test. Depending on given or not given homogeneity of variances, one-way Analysis of variance (ANOVA) or Welch’s ANOVA were performed for comparisons of mean values and Tukey-Kramer-Test or Games-Howell-Test were used for post-hoc analysis. To confirm results regardless of given normal distribution of data, Kruskal-Wallis-Test with direct comparisons adjusted by the Bonferroni correction was used. To compare nominal data, Fisher’s exact test was used. Receiver operating characteristic (ROC) curve was used to calculate a cut-off value with its associated sensitivity and specificity for OCTA.

An a priori power analysis was conducted using G*Power version 3.1.9.2 [30] for sample size estimation. Because no preliminary data about the subject of the study were available at the time of power analysis, the effect size was considered to be 0.4, which, according to current consensus, is a large effect for the ANOVA. With a significance criterion of α = 0.05 and power = 0.80, the minimum sample size needed with this effect size is *n* = 66, which equals *n* = 22 for each of the three cohorts. We did reach that number for the non-ICU- and the control group, but not for the ICU group.

## Results

The data of 70 eyes of 35 non-ICU patients, 10 eyes of 5 ICU patients and 74 eyes of 37 control patients were used for analysis. On four eyes of the non-ICU group (5.7%) and six eyes of the control group (8.1%), slit-lamp- and fundus examination was not performed. Anterior and posterior segment did not show any pathologies, except from disc drusen (5.7% of non-ICU group) and parapapillary atrophy (1.4% of non-ICU group). Mean intraocular pressure compared among all cohorts did not show a statistically significant difference (*p* = 0.156) (Table 1).

**Table 1:** Demographic and ocular characteristics of the study population

	Non-ICU group (n = 35)	ICU group (n = 5)	Control group (n = 37)	*p* value
Age (mean ± SD)	45.9 ± 11.5	51 ± 23.9	47.5 ± 12.5	0.618†
Sex				
Male (%) Female (%)	9 (25.7%) 26 (74.3%)	3 (60%) 2 (40%)	10 (27%) 27 (73%)	0.297‡
Days from PCR (± SD)	160 ± 47	76 ± 25		
Anterior segment* (%)				
Physiological Missing values	66 (94.3%) 4 (5.7%)	10 (100%) -	68 (91.9%) 6 (8.1%)	
Posterior segment* (%)				
Physiological Missing values Other	61 (87.1%) 4 (5.7%) 5 (7.1%)	10 (100%) - -	68 (91.9%) 6 (8.1%) -	
Intraocular pressure* (mean ± SD)	15.6 ± 2.1	14.5 ± 3.3	14.7 ± 3.4	0.156†

* Both eyes were used for analysis

† Welch’s ANOVA

‡ Fisher’s exact test

**Fig. 1:**
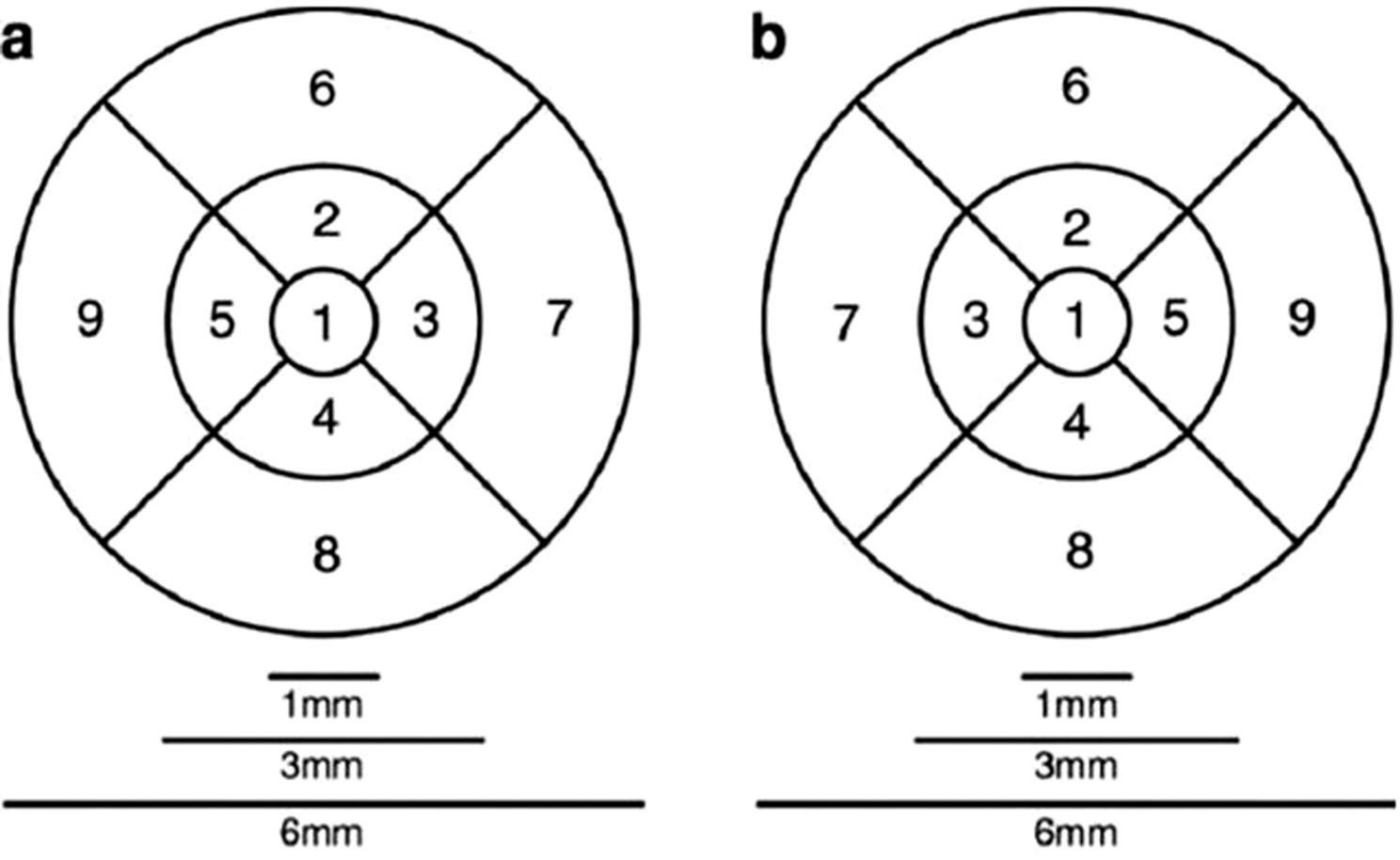
ETDRS grid. Nine ETDRS subfields in each eye. (**a**) Right eye. (**b**) Left eye.×. * reproduced from Demirkaya et al., 2013, *invest ophthalmol vis Sci*, 54(7):4934–4940, licensed under cc by 4.0 [31]

The comparison of mean total retinal thickness of the nine Early Treatment of Diabetic Retinopathy Study (ETDRS) grid subfields (Fig. 1) among the three cohorts did not report a statistically significant result (*p* = 0.345). The mean retinal thickness of each of the nine ETDRS grid subfields was also compared among all three cohorts, which presented no statistically significant differences (Table 2).

**Table 2:** Mean retinal thickness (µm) for disease groups

	Non-ICU group (n = 70)	ICU group (n = 10)	Control group (n = 74)	p value
Total* (± SD)	2811.9 ± 134.1	2870.5 ± 91.8	2829.6 ± 122.4	0.345†
ETDRS grid zone 1 (± SD)	277.1 ± 21.3	278.8 ± 15.6	280.2 ± 13.8	0.593‡
ETDRS grid zone 2 (± SD)	342.4 ± 16.8	348.3 ± 12.2	342.4 ± 16.4	0.545†
ETDRS grid zone 3 (± SD)	343.8 ± 17.4	348.4 ± 10.3	345.0 ± 15.9	0.691†
ETDRS grid zone 4 (± SD)	338.7 ± 17.2	346.0 ± 11.3	338.3 ± 18.6	0.422†
ETDRS grid zone 5 (± SD)	330.8 ± 16.7	337.4 ± 10.8	331.0 ± 15.0	0.447†
ETDRS grid zone 6 (± SD)	297.1 ± 15.8	302.9 ± 10.7	299.9 ± 17.3	0.428†
ETDRS grid zone 7 (± SD)	311.6 ± 17.2	317.9 ± 12.5	313.6 ± 18.1	0.519†
ETDRS grid zone 8 (± SD)	286.6 ± 16.0	295.9 ± 11.1	291.0 ± 21.0	0.175†
ETDRS grid zone 9 (± SD)	283.7 ± 16.5	294.9 ± 10.9	285.5 ± 13.7	0.086†

* Of the nine ETDRS grid subfields

† ANOVA

‡ Welch’s ANOVA

For each parameter, both eyes were used for analysis

**Fig. 2:**
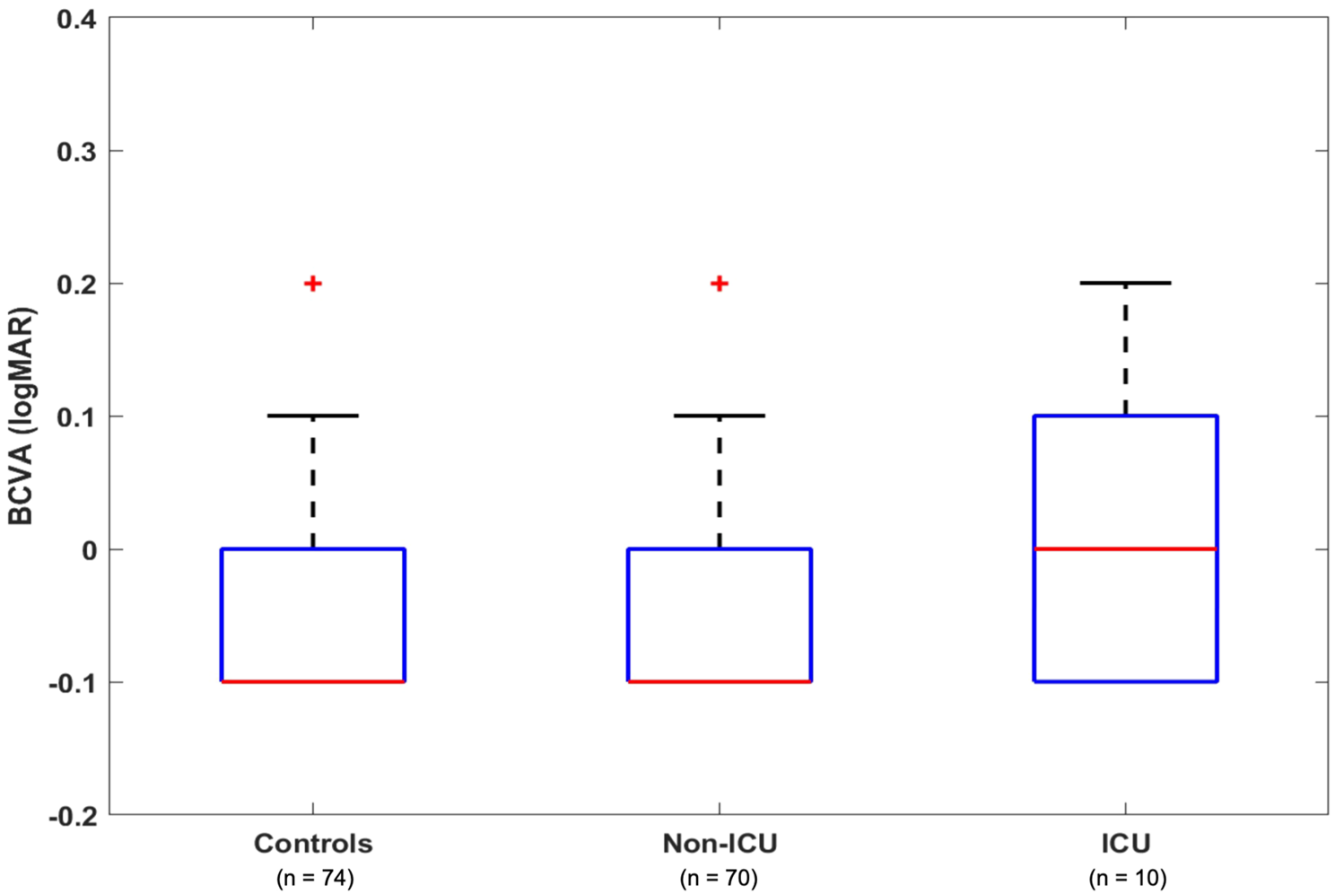
BCVA, mean with SD, Welch’s ANOVA, *p* = 0.116

Comparing the mean best-corrected visual acuity (BCVA) among all three cohorts, there was no statistically significant difference (*p* = 0.116). Though that difference did also not reach statistical significance (*p* = 0.141), mean BCVA of the ICU group was slightly lower than of the control group (Fig. 2).

**Fig. 3:**
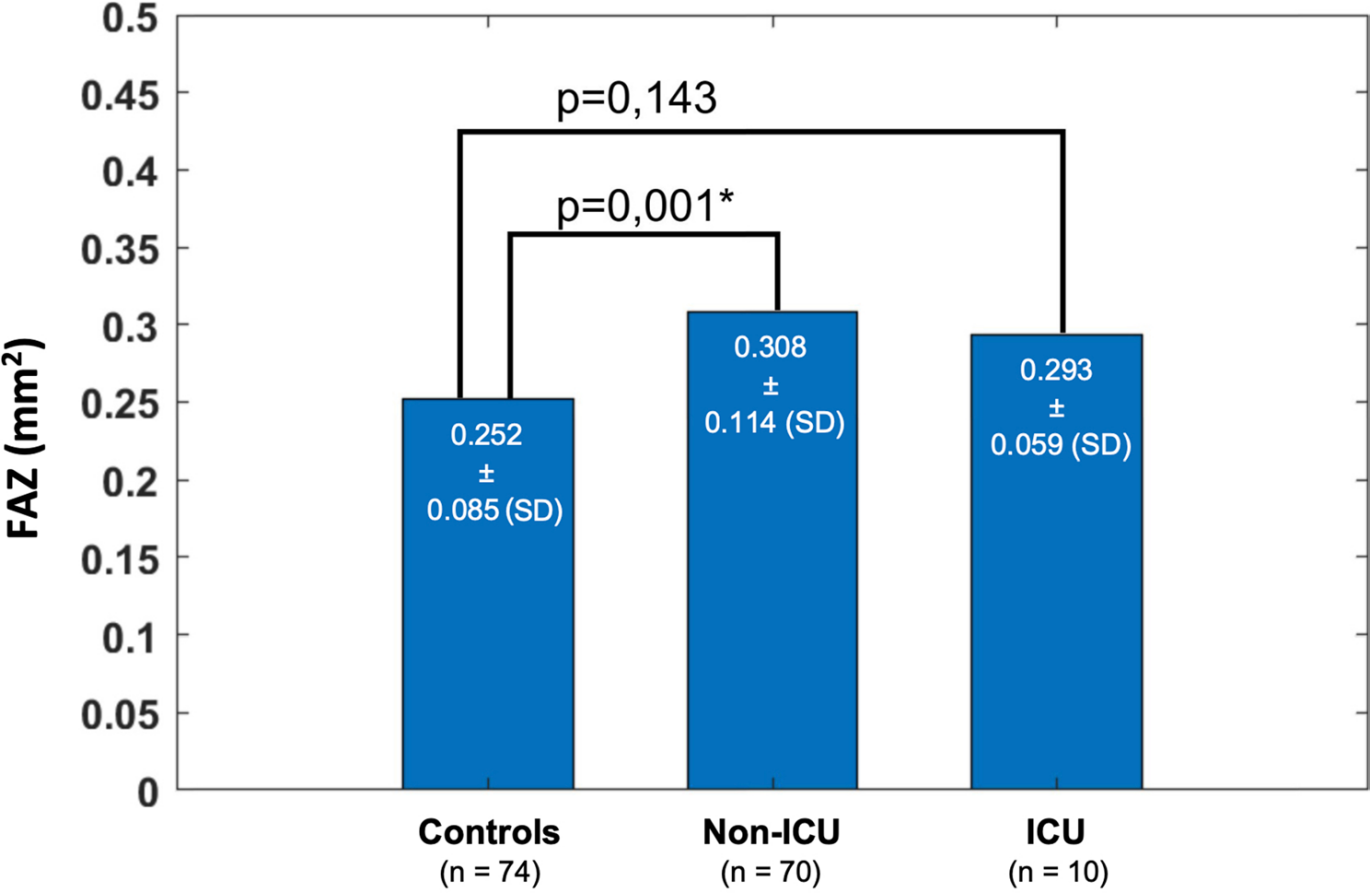
FAZ, mean with SD, Welch’s ANOVA, *p* = 0.006*

**Fig. 4:**
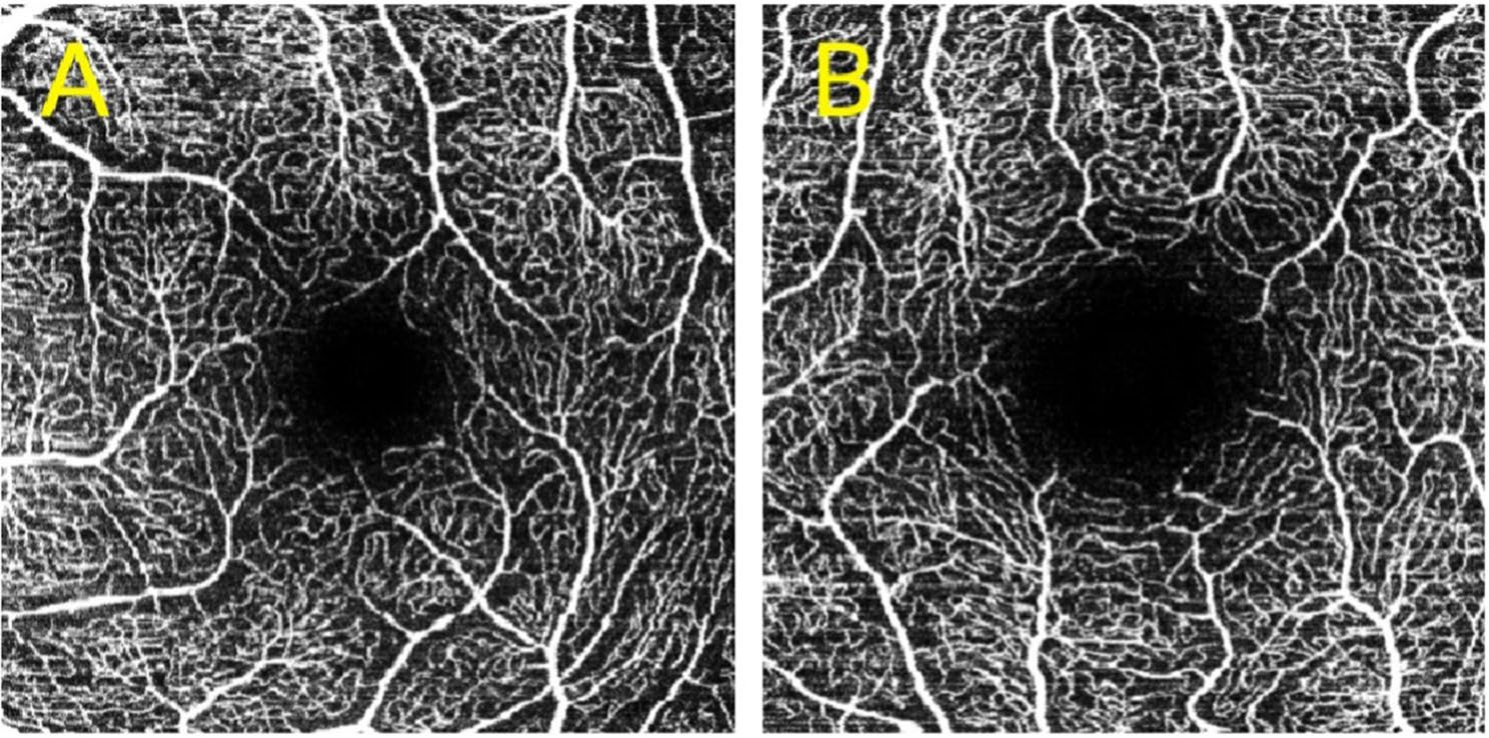
OCTA image of SCP of control patient (**A**) and non-ICU patient (**B**)

FAZ area showed a statistically significant difference among the three cohorts (*p* = 0,006). Mean FAZ area of the non-ICU group was greater than mean FAZ area of the control group (*p* = 0.003). Mean FAZ area of the ICU group showed a trend towards being larger than control FAZ, however not significant (*p* = 0.163) (Figs. 3 and 4).

**Fig. 5:**
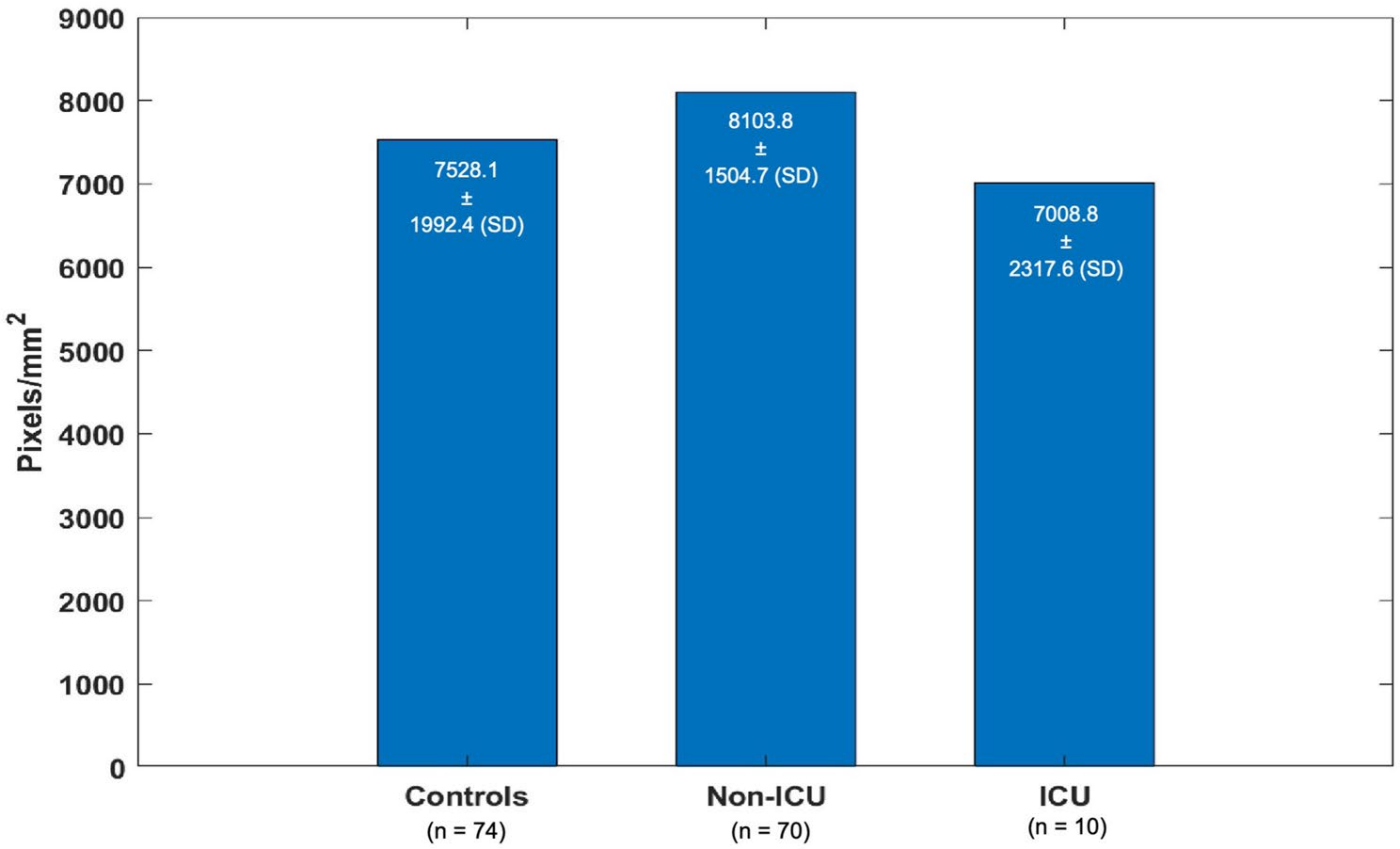
VD, mean with SD, Welch’s ANOVA, *p* = 0.099

Mean VD among the three cohorts was not significantly different (*p* = 0.099). Mean VD of the non-ICU group and mean VD of the ICU group did not differ significantly from mean VD of the control group (Fig. 5).

Subdivision of patients regarding their fatigue symptoms includes 14 subjects with no fatigue, 17 subjects with fatigue, 6 subjects with extreme fatigue and 37 control subjects (FAS-Score).

FAZ revealed to be significantly different comparing the mean FAZ area among the four cohorts with the ANOVA (*p* < 0.001). In post-hoc analysis, a significantly enlarged FAZ of patients with fatigue in comparison to patients with no fatigue (*p* = 0.018) and to the control patients (*p* < 0.001) could be seen.

**Fig. 6:**
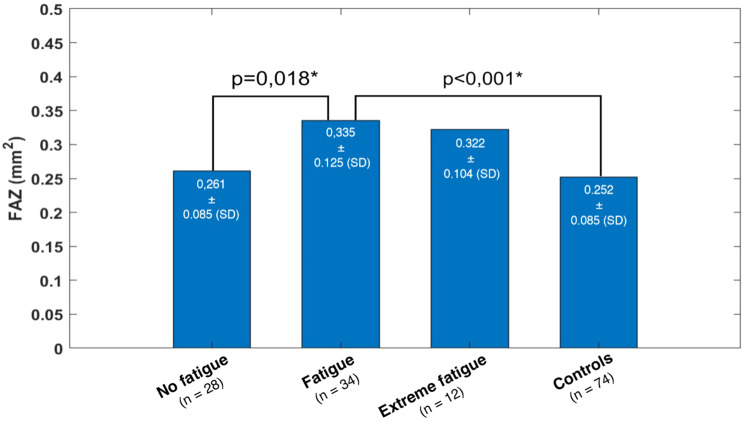
FAZ, mean with SD, ANOVA, *p* < 0.001*

**Fig. 7:**
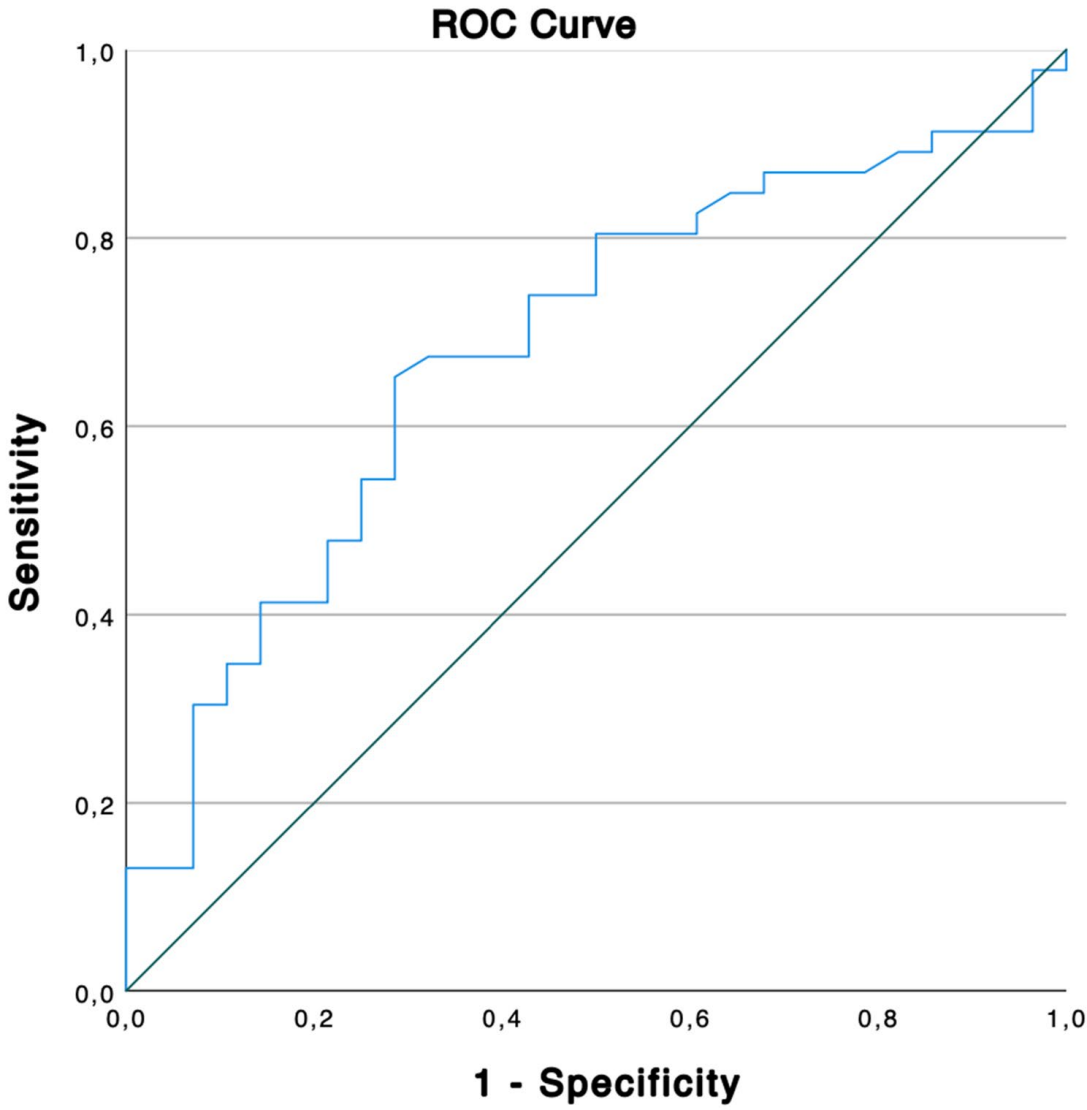
ROC curve of patients with fatigue (*n* = 46 eyes)

Based on the ROC curve using FAZ of all patients with any expression of fatigue (*n* = 46 eyes), the sensitivity of OCTA for fatigue detection is 65.2% and specificity is 71.4%, given the cut-off value of 0.291 mm^2^ for FAZ. Together with the prevalence of 23% of fatigue within the subgroup of all those formerly infected with SARS-CoV-2 [13], this results in a positive predictive value (PPV) of 40.51% of OCTA for detecting fatigue by measuring FAZ area (Figs. 6 and 7). The ROC analysis yielded an area under the curve (AUC) of 0.683 (95% CI: 0.56–0.80).

**Fig. 8:**
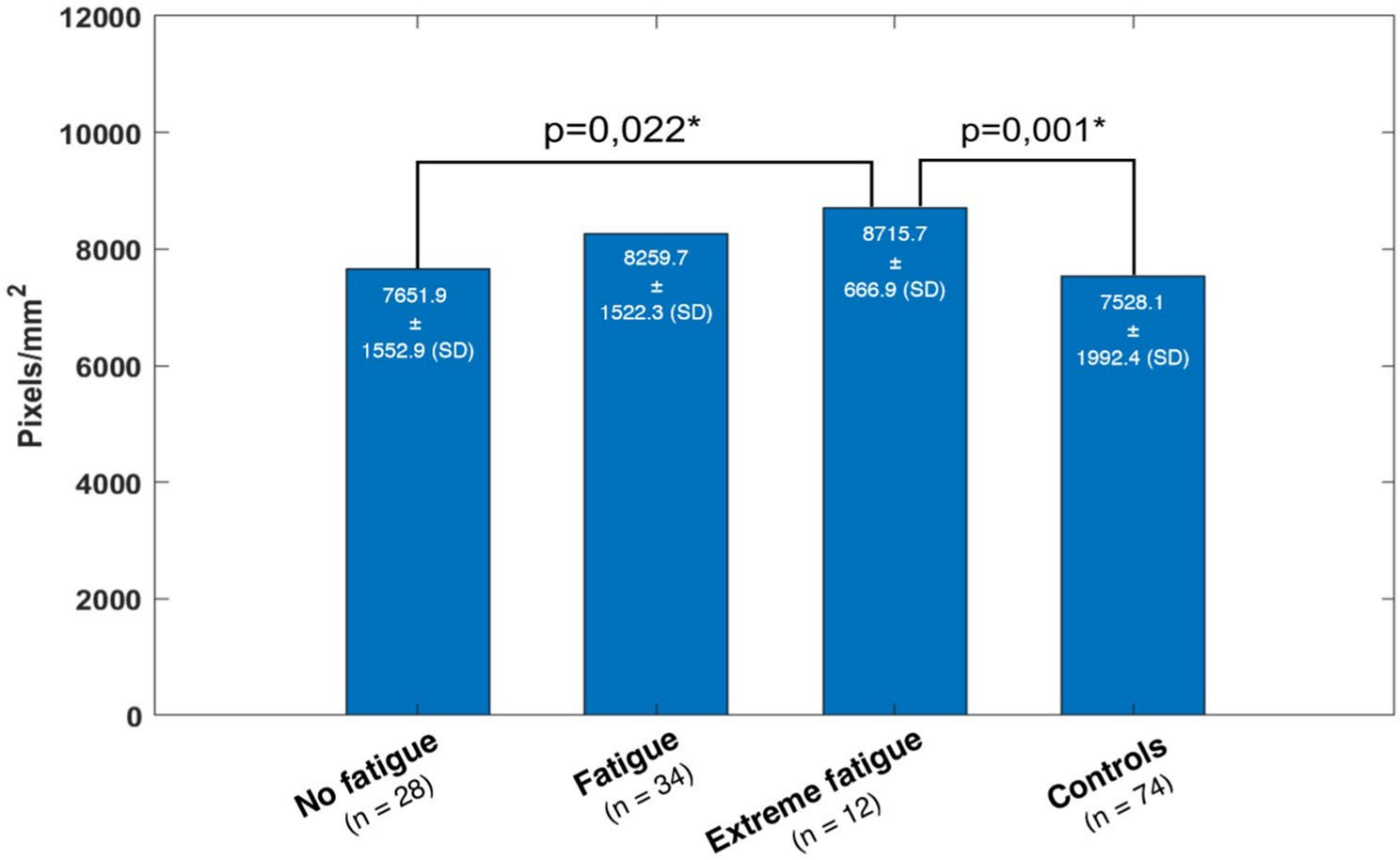
VD, mean with SD, Welch’s ANOVA, *p* = 0.001*

The comparison of VD of the fatigue subgroups also showed a statistically significant difference (*p* = 0.001). The group of patients with extreme fatigue showed a greater VD than the group of patients with no fatigue (*p* = 0.022) and the control group (*p* = 0.001) (Fig. 8).

Mean BCVA, intraocular pressure and total retinal thickness compared among all fatigue subgroups did not show statistically significant differences.

**Fig. 9:**
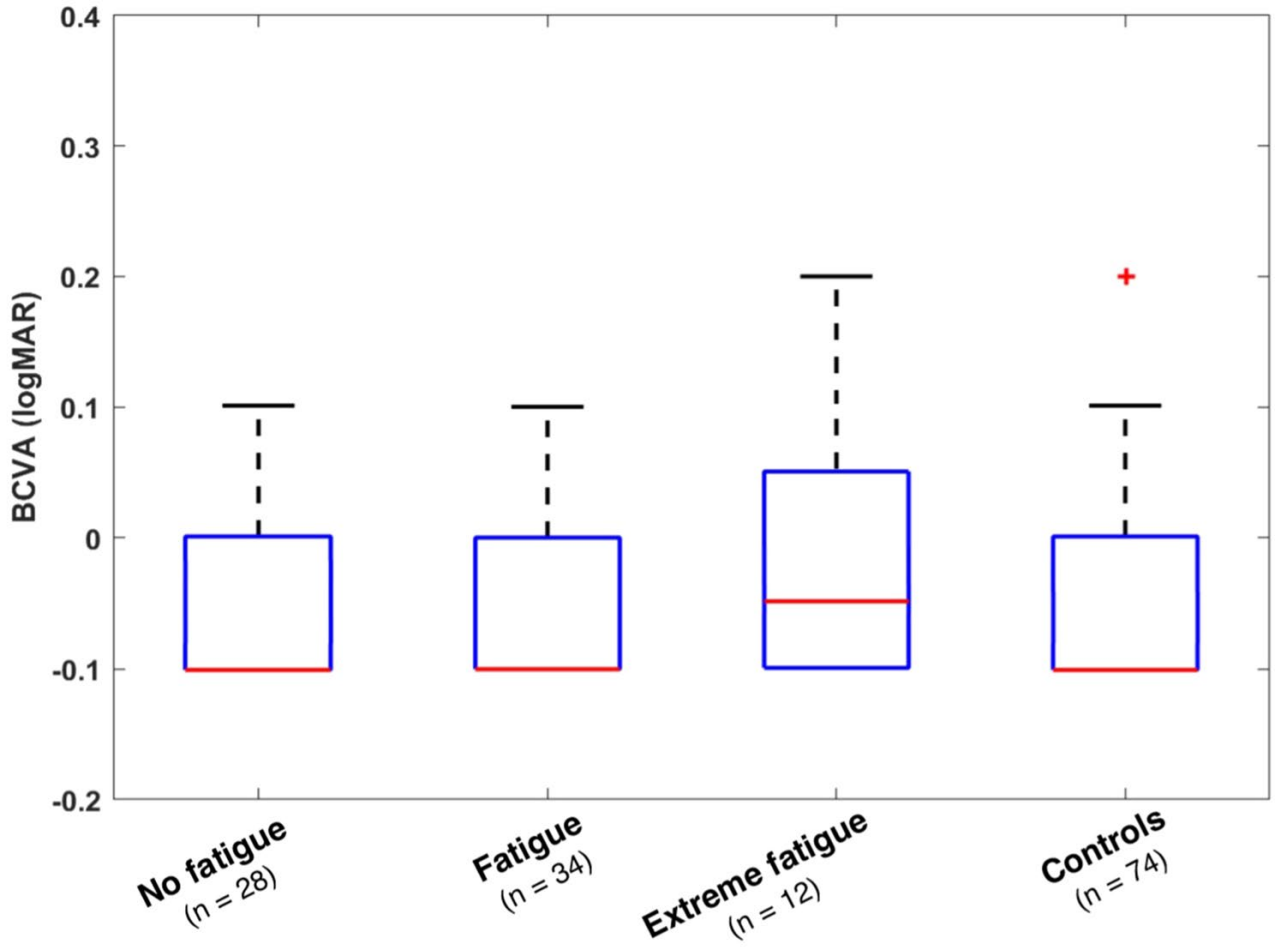
BCVA, mean with SD, Welch’s ANOVA, *p* = 0.337

Mean retinal thickness of the ETDRS grid subfields 1 to 5 also did not differ statistically significant among these subgroups, but for subfields 6 to 9 such a statistically significant difference could be seen in each. In each of these subfields, post-hoc analysis showed a significantly lower retinal thickness of the patients with no fatigue compared to the patients with fatigue and to the control patients (Fig. 9) (Table 3).

**Table 3:**
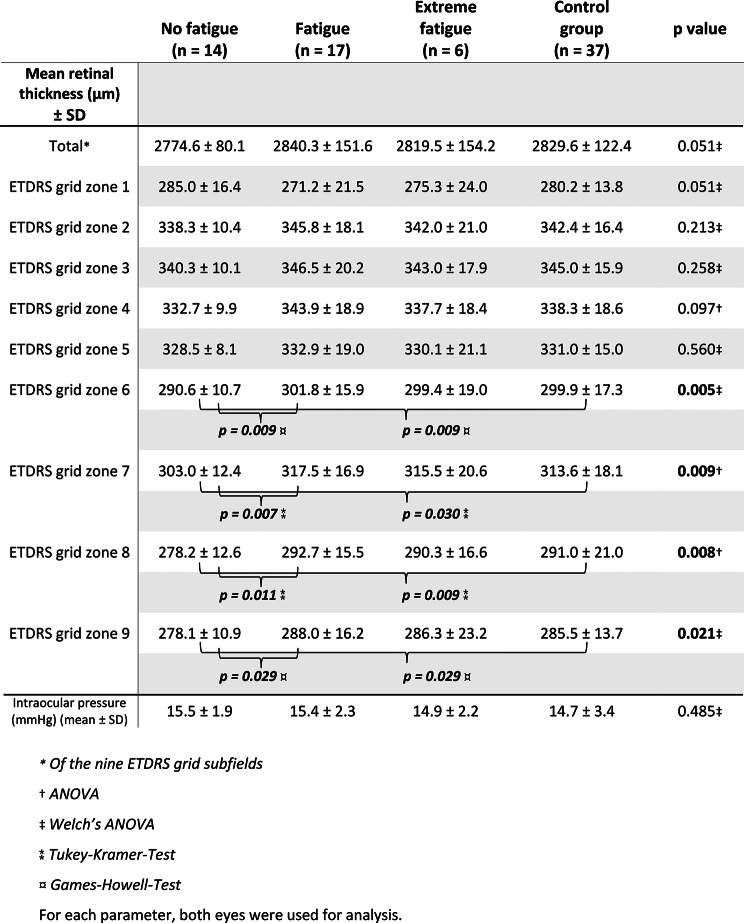
Retinal thickness (µm), intraocular pressure for fatigue groups

## Discussion

Our study describes microvascular alterations in the sense of a significantly enlarged FAZ in COVID-19 patients and patients with fatigue due to long COVID-19 compared to the control group of similar age and sex distribution. The VD of the SCP was not significantly altered, just as the BCVA and retinal thickness in the macular region.

SARS-CoV-2 is thought to use ACE2 as a receptor to enter host cells. In addition to that, transmembrane protease serine subtype 2 (TMPRSS2), a cell-surface-associated protease, facilitates viral entry after the spike-protein binds to ACE2 [32]. Both ACE2 and TMPRSS2 are expressed in various retinal layers and the retinal endothelium and also in the SCP that is analyzed in our study [7, 23]. The virus then causes cellular dysfunction and dysregulated inflammatory and coagulation processes in the vasculature [33]. At the same time, SARS-CoV-2 induces platelet hyperactivation, followed by inflammation, endotheliopathy and sometimes thrombosis in large and small vessels [34], which in combination can be referred to as thromboinflammation. Other studies consider this combination of direct non-ischemic damage and thromboinflammation to be responsible for reported retinal microvascular alterations [25, 35], and our results may also be influenced by these factors.

The systematic review of current evidence by Teo et al. showed reduced parafoveal VD and increased FAZ in COVID-19 patients [24]; a more recent meta-analysis by Kazantzis et al. reported an increased FAZ as well as reduced foveal, parafoveal and whole image VD in deep capillary plexus (DCP) compared to healthy controls [36].

Another study reported long-term persistence of these changes for up to 8 months [37]. However, in our study only the increased FAZ was statistically significant. There is a probable association between lower VD and larger FAZ with COVID-19 subjects compared to controls [24], suggesting that the changes in each of the two parameters allow a similar assessment of the state of the retinal microvasculature.

Unlike in other tissues, retinal plexi do not form anastomoses, but are composed of end arteries [38]. So, the FAZ boundaries are formed by such end arteries, which might cause particular susceptibility for ischemia [39] and could in conclusion be a possible explanation for the seen alterations of FAZ.

In diabetic retinopathy, an enlarged FAZ is also frequently seen due to ischemia and is often associated with worsening visual acuity and may indicate progression of the underlying disease [40–42]. In cases of retinal vein occlusion and moderate-advanced glaucoma, an association between an enlarged FAZ and reduced visual acuity has also been demonstrated [43, 44]. Moreover, in glaucomatous eyes, a significantly enlarged FAZ was observed in patients with central visual field defects compared to those with peripheral visual field defects [45].

However, to date, most available studies on retinal microangiopathy following COVID-19 infection have not reported significant changes in visual acuity [26, 27, 35]. It should be noted that several investigations focusing on OCTA parameters did not include an analysis of BCVA. Accordingly, meta-analyses have found no significant reduction in visual acuity among COVID-19 patients with retinal microangiopathic alterations [24, 36]. These meta-analyses, however, emphasize the need for longitudinal studies to determine whether microvascular changes persist over time and to assess potential clinical implications, such as reduced visual acuity or other visual symptoms.

Similarly, in our study the patients did not report subjective visual impairment at the time of examination, and BCVA did not differ significantly between the cohorts.

Still, the enlarged FAZ that could be seen, may represent a potential risk of developing ophthalmologic complications such as impaired vision in the future. Longitudinal studies of such patients will be needed to gain further insight in this regard.

The OCTA technique, which we used to obtain our results, is an easy way to examine the microvascular status of subjects, as it is non-invasive, easy to handle and provides reliable and detailed information about retinal vessels [28]. Though only retinal microvasculature can be seen, OCTA parameters may reflect the microvascular status of other organ systems, as well [46–50]. Accordingly, Cosmo et al. found that a prior cardiac or cerebrovascular event had a negative impact on the macular microvasculature in patients with COVID-19, while systemic hypertension showed a weaker association [51]. Patients with diabetic retinopathy were found to have an increased risk of an adverse outcome from COVID-19 infection [52], further highlighting the bidirectional relationship between the retinal microvasculature and the microvascular status of other organ systems.

Particularly the microvascular status of other neuronal tissues can be evaluated in an easy way using OCTA, because the retinal and cerebral microvasculature show an anatomical and physiological homology [38]. This is especially relevant as there are currently no clinical applicable imaging methods that allow for direct, non-invasive measurement of cerebral microvasculature [53]. Evidentially, retinal microvascular changes reflect cerebral microvascular changes not only in aging, but also in neurological diseases as vascular dementia, stroke, and others [38, 47, 54]. This association also appears to be relevant in the context of COVID-19, as SARS-CoV-2 is thought to induce microangiopathy in neural tissues as well. In a post-mortem study of COVID-19 patients, neuropathological examination revealed cerebral and cerebellar ischemic lesions, with perivascular inflammation associated with hypoxic neuronal damage [55].

Having that in mind, in the future it will be important to investigate whether retinal and cerebral microvascular changes are related in patients with COVID-19.

In our study, a significantly increased FAZ was shown for the group of patients with fatigue compared to the patients with no fatigue and to the control group. There are indications that in long COVID-19, after the stage of acute SARS-CoV-2 infection, persisting endotheliopathy with thromboinflammation is significantly involved in the pathogenesis of fatigue [56], with findings of consistently exhibited inflammatory markers in some long COVID-19 patients suffering from fatigue [14]. Furthermore, there is general consensus on a causal relationship between inflammation in terms of the presence of specific inflammatory cytokines and mood disorders and cognitive impairment [57, 58]. Considering the mentioned important role of inflammation and endotheliopathy in the pathogenesis of retinal microangiopathy after COVID-19, these mechanisms seem to be possibly linked with the occurrence of an enlarged FAZ in patients with fatigue. Schlick et al. recently showed the association between fatigue and retinal microvascular impairment in post-COVID-19 patients using OCTA [59]. In attempt to investigate whether OCTA can be used as a predictor of long COVID-19, we calculated a PPV of 40.51% of OCTA for detecting fatigue by measuring FAZ area. Though fatigue is the most common symptom in long COVID-19 condition, its prevalence of only 23% within all formerly SARS-CoV-2 infected results in this relatively low PPV. We also calculated the AUC of 0.683 (95% CI: 0.56–0.80), which is indicating a poor to moderate discriminative ability of FAZ area to differentiate between patients with and without fatigue [60]. However, the significance of these values is limited given the small sample size and needs further validation in future studies but provides a potential association between FAZ, microvascular impairment and fatigue.

Our results show the increased FAZ only for the patients with fatigue, but not for those with extreme fatigue. This may be due to the small number of patients with extreme fatigue (*n* = 12 eyes), which may have prevented statistical significance of a possible effect. Nevertheless, the tendency of an increased FAZ is also present in this cohort.

Contrary to what would be expected, our study shows a significantly greater VD in the patients with extreme fatigue compared to the patients with no fatigue and to the control group. A result to be expected would be an increased FAZ correlating with a decreased VD; also, in the study of Schlick et al. a decreased VD was found for different subgroups of post-COVID-19 syndrome patients, but no increased VD for any subgroup [59]. Pathophysiologically, no conclusive explanation can be found for that increased VD, so that it must also be discussed whether this is due to the small number of cases in the group of patients with extreme fatigue and could be seen as a statistical outlier.

Another finding of our study is significantly lower retinal thickness in the perifoveal region (ETDRS grid subfields 6–9) of the patients without fatigue compared to patients with fatigue and to healthy controls. Since ACE2 receptor, used by SARS-CoV-2 to enter host cells, is expressed in multiple nonvascular neuroretinal cells, including the retinal ganglion cell layer, inner plexiform layer, inner nuclear layer, and photoreceptor outer segments [23], not only the retinal microvasculature, but also retinal thickness represents a relevant parameter in investigating retinal alterations linked to COVID-19. Sumer and Subasi reported thinning of the temporal and superior perifoveal regions following COVID-19 infection [61], whereas findings regarding the central macular thickness (CMT) have been inconsistent, with studies describing it as thinned, thickened, or not significantly altered [21, 62, 63]. In this context, the reduced perifoveal retinal thickness observed in patients without fatigue compared to healthy controls in our study might be associated to previous COVID-19 infection. Sumer and Subasi discuss whether transient macular thickening due to inflammation in the acute stage may be followed by atrophic changes over time, just as in other neurodegenerative diseases [61]. Longitudinal studies with larger sample sizes are required to verify this association, which has so far been discussed on a theoretical basis.

However, given the current state of research, a reduced retinal thickness in patients without fatigue compared to patients with fatigue would not be an expected finding. This observation may be related to the small sample size and could represent a statistical outlier.

There are some limitations to our study. Due to the absence of baseline data, a direct causal link between COVID-19 infection and retinal microvascular alterations cannot be established. Previous retinal damage was an exclusion criterion for study participation, but unpredictable confounding factors, such as undiagnosed cardiovascular comorbidities or undiagnosed pre-existing fatigue, may have contributed to the observed alterations or influenced the individual susceptibility to microvascular damage. Therefore, the observed enlargement of the FAZ should be interpreted as an association rather than a direct consequence of the infection. Future longitudinal studies, ideally including baseline data, are required to provide further insight into the development of these alterations. Moreover, the small sample size of both the ICU patient group in comparison of the COVID-19 status subgroups and the group of patients with extreme fatigue in comparison of the fatigue subgroups should be mentioned, which could have either masked statistically significant results or lead to unexpected results. Furthermore, no objective, software-based quality scan of the OCTA images was performed, so artifacts in the images may have caused inaccuracies in the measurement of VD, which could also be a possible explanation for the observed increased VD in the subgroup of patients with severe fatigue. OCTA images deemed unsuitable due to artifacts were manually excluded, but without masked review or adjudicated assessment.

As we focused on macular OCTA images using a field of view of 3 × 3 mm, this can be seen as another limitation to our study, as microvascular impairment more peripheral than that could not be detected.

It must also be discussed that the Shapiro-Wilk test did not show a normal distribution for all data in our study. However, the Kruskal-Wallis-Test with direct comparisons adjusted by the Bonferroni correction as a non-parametric test, which generally does not require a normal distribution of data, also confirms the main results of the study. Thus, the FAZ of the non-ICU patients is also significantly greater than that of the control group; moreover, the FAZ of the patients with moderate fatigue is also significantly greater than the FAZ of the patients with no increased fatigue and the FAZ of the control group. There is evidence that the ANOVA as a parametric test is nevertheless relatively robust to violations of the normal distribution of data [64, 65], so that in the following we continued to use the ANOVA and consecutive post-hoc tests, evaluating and discussing these results.

Furthermore, the control group subjects were considered to have no history of SARS-CoV-2 infection based only on the anamnesis, so SARS-CoV-2 infection in the past could not be excluded with complete certainty. However, in the period of patient recruitment (July 2020 to August 2021), there were still relatively low levels of COVID-19 incidence in the general population compared to later stages of the pandemic [66], so therefore, we do not consider this limitation of the study to be of great importance.

## Conclusions

Our study demonstrates an association between COVID-19 disease and retinal microvascular alterations, reflected by an enlarged FAZ, especially in non-ICU patients and in long COVID-19 patients with fatigue, highlighting the potential global health relevance given the high number of mild cases of COVID-19 and of long COVID-19 patients. To our knowledge, this is one of the first studies to report an association between retinal microvascular alterations, COVID-19 disease severeness (ICU) and fatigue in long-COVID-19 patients.

In the future, longitudinal studies with larger sample sizes are required to evaluate the possible progression of retinal microangiopathy in patients, for which OCTA is a suitable, non-invasive tool. Furthermore, OCTA might serve as a predictor for long COVID-19 disease, a hypothesis that requires further validation by studies with larger sample sizes.

## Data Availability

The datasets used and/or analysed during the current study are available from the corresponding author on reasonable request.

## Abbreviations

ACE2: Angiotensin-converting enzyme
ANOVA: Analysis of variance
AUC: Area under curve
BCVA: Best-corrected visual acuity
CMT: Central macular thickness
COVID-19: Coronavirus disease 2019
DCP: Deep capillary plexus
ETDRS: Early Treatment of Diabetic Retinopathy Study
FAS: Fatigue Assessment Scale
FAZ: Foveal avascular zone
ICU: Intensive care unit
MHH: Hannover Medical School
NICE: National Institute of Health and Care Excellence
OCTA: Optical coherence tomography angiography
PPV: Positive predictive value
ROC: Receiver operating characteristic
SARS-CoV-2: Severe acute respiratory syndrome coronavirus type 2
SCP: Superficial retinal capillary plexus
TMPRSS2: Transmembrane protease serine subtype 2
VD: Vessel density
WHO: World Health Organization
